# Predictors of vitamin A rich food consumption among women living in households growing orange-fleshed sweetpotatoes in selected regions in Uganda

**DOI:** 10.3389/fpubh.2022.880166

**Published:** 2023-01-09

**Authors:** Joyce Nankumbi, Frederick K. E. Grant, Lindiwe Sibeko, Evelyn Mercado, Norman Kwikiriza, Simon Heck, Lorraine S. Cordeiro

**Affiliations:** ^1^Department of Nutrition, University of Massachusetts, Amherst, MA, United States; ^2^International Potato Center Kampala, Kampala, Uganda; ^3^Department of Psychological and Brain Sciences, University of Massachusetts, Amherst, MA, United States; ^4^International Potato Center (ILRI), Nairobi, Kenya

**Keywords:** vitamin A, women, Uganda, food consumption, knowledge

## Abstract

**Objective:**

Vitamin A deficiency (VAD) has serious public health consequences including morbidity and mortality for populations in low and middle-income countries (LMICs), especially for children under 5 years and pregnant women. LMICs are at greater risk of VAD, in part due to low levels of consumption of vitamin A-rich foods most of which are plant-based, such as orange-fleshed sweet potatoes (OFSP), with lower bioavailability than animal sources of the vitamin A. Food-based approaches such as biofortification of OFSP, including promoting the consumption of vitamin A-rich biofortified staple crops, has been shown to be potentially effective in improving the status of vitamin A and other micronutrients. This study examined vitamin A-rich food consumption and its predictors among women of reproductive age from OFSP-growing households in two regions of Uganda.

**Methods:**

A cross-sectional survey was conducted among 617 OFSP growing households, focusing on women in the reproductive age group from the northern and eastern regions of Uganda. Households were not receiving any VAD-related intervention at the time of the survey. Quantitative data included vitamin A-rich food consumption, knowledge on vitamin A, and rich food sources dietary intake, using a 7-day food frequency questionnaire. Vitamin A consumption and risk of deficiency were estimated using the Hellen Keller International guide.

**Results:**

The majority of women in this study were either pregnant (80%) or lactating (17%). More than 70% of the study population had a weighted vitamin A rich food consumption mean score of <6 days per week, indicating a high risk of VAD. Knowledge about vitamin A [b (SE) = −0.18 (0.50), *p* < 0.001] was significantly and inversely associated with vitamin A rich food consumption.

**Conclusion:**

Components of food insecurity such as availability, affordability, utilization, and changing food preferences may contribute to the unexpected inverse relationship between knowledge and consumption of vitamin A rich foods. Scaling up biofortified food initiatives, including OFSP, can improve consumption of vitamin A rich foods with effective strategies to comprehensively address consumption barriers such as lack of nutrition education, cooking skills, and storage facilities, as well as low production levels and perceived contamination of biofortified foods.

## Introduction

Micronutrient deficiencies, especially vitamin A, iron, folate, and iodine, are highly prevalent in low- and middle-income countries (LMIC) (1, 2). Micronutrients contribute to poor health (3), retarded growth, low productivity, growth impairment, and unexpected death (4–7). The burden of disease due to the health effects of micronutrient deficiencies has received increased attention in the last few decades (3). Women and children are at a higher risk of developing micronutrient deficiencies, with pronounced effects during preconception, pregnancy, and lactation (8, 9). These conditions are aggravated by low levels of household dietary diversity (4) and household food insecurity (10).

Vitamin A deficiency (VAD) is considered one of the most serious public health concerns in LMIC. Young children and pregnant women, especially in low-income, rural communities, are more susceptible to VAD. Vitamin A is an essential micronutrient for normal functioning of the visual system, growth, and development, as well as maintenance of epithelial cellular integrity, immune function, and reproduction (11). VAD, defined as serum retinol concentrations below 0.825 μmol/l (12), is estimated to affect two billion people globally (7, 13). The initial symptoms of VAD include impaired adaptation to the dark with serum retinol concentrations falling below 0.8 μmol/L (14). Xeropthalmia, an advanced condition of VAD that can lead to blindness, is a concern for pregnant women given greater vitamin A requirements during this period of the life course (7).

Globally, evidence supports dietary diversification for optimal nutrition and health (15, 16). Consumption of diverse diets is central to achieving and preserving nutrient adequacy throughout the life course (15, 16). Inadequate dietary quantity and quality result in deficiencies of essential nutrients, particularly during pregnancy and lactation (17). In China, inadequate consumption of fruits and vegetables was associated with low levels of serum vitamin A among lactating women (18). In Ethiopia, more than 70% of pregnant women had low dietary diversity scores and low consumption of animal sourced foods (19) and 60% of lactating women had inadequate consumption of vitamin A rich foods (20). Diets of healthy women of reproductive age in Indonesia were considered low in animal sourced foods and fruit and vegetable consumption, suggesting greater risk of nutrient deficiencies (21). Understanding barriers to consumption of vitamin-A rich foods is critical to food-based programming, especially in post conflict regions of the world which are prone to drought and other conditions that exacerbate food insecurity.

Despite the progress in addressing vitamin A deficiency in LMICs, including Uganda, it is still a persistent public health concern. In Uganda, the prevalence of VAD is 8.3 and 9.0% in the urban and rural areas, respectively (22). Higher prevalence rates have been reported in the following subregions of Uganda: Acholi (15.4%), Busoga (12.8%), and West Nile (11.2%) (22). High implementation costs of industrial food fortification and supplementation programs, and their limited reach in resource-poor rural communities, justify continued support of alternative food-based approaches such as diet diversification and the biofortification of daily staple crops, such as orange-fleshed sweet potatoes (OFSP), with vitamin A (23). Innovative food-based approaches have included production of beta-carotene rich OFSP as a preventive and therapeutic nutrition-specific intervention targeting livelihoods that can also potentially prevent and mitigate vitamin A deficiency (24).

More research is needed on dietary patterns related to consumption of vitamin A rich foods, including OFSP, among women during pregnancy and the postpartum period (25, 26). A better understanding of dietary patterns, including vitamin A rich food consumption such as intake of OFSP, can lead to improved interventions to prevent malnutrition and improve maternal and child health outcomes related to VAD and other micronutrient deficiencies. Examination of dietary patterns also has implications for dietary guidelines (27), which can subsequently lead to increased understanding of the mechanisms that facilitate higher consumption of vitamin A rich foods and improved vitamin A status among women. This study examined consumption patterns of vitamin A rich foods, including OFSP, and assessed the predictive capacity of knowledge of vitamin A rich foods, knowledge of OFSP, and misconceptions of OFSP on vitamin A rich food consumption among women from OFSP-growing households in two regions of Uganda.

## Methods

### Study design

This study examined baseline data collected in 2020 on dietary intake and livelihoods among 617 women residing in households growing OFSP from two regions in Uganda. Survey data was collected by the International Potato Center (CIP) as a baseline for their “Development and Delivery of Biofortified crops to scale (DDBIO)” project. Uganda is a landlocked country in East Africa with an estimated population of 43 million people (28). More than half (50.8 %) of the Ugandan population are women and nearly seven million are < 4 years of age (22). With 74% of the population residing in rural areas, the agricultural sector is the major economic backbone for the nation's population (29). The country is divided into four regions: central, western, eastern and northern, and these comprise of subregions of a number of districts. This study focused on data collected by CIP in 2020 in nine out of the twelve districts considered for the DDBIO project including Pader, Lamwo, Gulu, Kitgum, Agago, and Adjumani in the northern region and Busia, Tororo, and Karamoja in the eastern region of Uganda.

### Study population

The study population comprised women who resided in households that grew sweet potatoes in the eastern and northern regions of Uganda. Districts within these regions were selected based on relatively higher prevalence of malnutrition as compared to other districts in the country, and limited efforts to improve nutritional status *via* food-based interventions. Baseline survey participants were recruited from the communities at the household level. Eligible participants were women in each of the households who were either pregnant or lactating, as well as adolescent girls. Women were ineligible to participate in the survey if they were sick or mentally unwell or had not stayed in the household in the past 3 months.

The sample size for this study was determined based on a priori sample size calculations. The statistical power needed to detect small, medium, and large effects was calculated for chi-square, *t*-tests, and regression analyses. Power analyses were conducted using G^*^ power 3.1.9.7 software (30). Assuming a two-tailed test, 1- beta error probability of 0.8, and an alpha of 0.05, a sample size of 617 was determined to be sufficient to detect medium and larger sized effects for all tests, and to detect associations between selected independent variables and the outcome variable, vitamin A rich food consumption.

### Data collection

Using a comprehensive semi-structured household-level survey, data included demographic characteristics, household food consumption, a 7-day food frequency questionnaire for vitamin A rich foods, knowledge about vitamin A, and other information. The survey tool was administered at the household level in local languages by trained research assistants.

### Measures

#### HKI food frequency method

An adjusted Hellen Keller International (HKI) guide was used to collect data on vitamin A rich food consumption. The HKI Food Frequency Method, which has been validated against WHO standards to classify VAD (31) is based on a 7-day food frequency questionnaire consisting of 33 food items to capture vitamin A food intake. Participants were asked, “How many days, in the past seven days, did (a selected reference child/woman) eat (a specific food item)?” The HKI method assesses the extent to which communities and populations are at risk of VAD. If at least 70% of the communities surveyed (11 out of 15) are assessed as having a high prevalence of VAD, vitamin A deficiency is likely to be a public health problem in the entire area. VAD status is determined by either of two threshold values: ≤ 4 days per week for mean frequency of consumption of animal sources of vitamin A or ≤ 6 days per week for mean frequency of total consumption of animal and plant sources of vitamin A (weighted by the source), or a combination of these (31, 32). The vitamin A consumption frequency score was using the following formula:

Weighted total consumption days (Cw) = Total number of days animal sources of Vitamin A consumed (TVA) + Total number of days plant sources of Vitamin A consumed (TAP) divided by 6.

The weighted vitamin A consumption score (C) is equal to the total number of days the mother consumed vitamin A rich foods from animal sources plus the adjusted consumption from plant sources.

#### Women's knowledge of vitamin A

Interviewers asked open-ended questions on knowledge of vitamin A rich foods and knowledge of OFSP (see Supplementary material). For vitamin rich foods, interviewers recorded participant responses by checking off each answer from a list of possible responses; a write-in option allowed interviewers to record unlisted foods. A score of one was allocated for each correct response. We summed up the individual responses of the section items; summed responses were on a scale of 0–5, with 0 representing a woman not having heard about vitamin A or with no correct answer for the rest of the items and five for a woman who has heard about vitamin A, knows two benefits of vitamin A and correctly identified two rich sources of vitamin A.

#### Women's knowledge of orange-fleshed sweet potatoes

Two scales were developed by running a series of exploratory factor analyses (i.e., principal axis factoring and direct oblimin rotation) to examine how many underlying factors explained women's knowledge of OFSP. Examination of the structural matrix allowed for the identification of items related to the underlying factors. Cut-off points of 0.32 were used for factor cross loading and 0.6 for a strong factor loading (33, 34). Items that had strong cross loadings or items that were shown to have factors that were not associated with knowledge, for example, responses to gender-related measures such as “sweet potato is a woman's crop and you can't grow sweet potatoes and be considered a man,” were eliminated. Two factors were identified in this analysis: Factor one (misconceptions about sweet potatoes) with items: (1) Sweet potato is not good for children less than 2 years old, (2) Sweet potato is not good for pregnant women, and (3) Sweet potato is not good for lactating women; and factor two (the general benefits of sweet potatoes) with items: (1) Sweet potato leaves are good for human beings to consume and (2) Sweet potatoes that are orange inside are healthier than the ones that are white inside. The reliability test for the two scales yielded a mean of 2.46 (SD 1.07) with a Cronbach's alpha of 0.362 for the general health benefit item and a mean of 3.90 (SD 0.831) with a Cronbach's alpha of 0.662 for the misconceptions about OFSP, respectively. Participants' responses to the generated items/scales were considered for comparison with vitamin A consumption.

#### Individual dietary diversity score

Household dietary diversity represents the number of different food groups consumed by the household within a specified recall period (35). The individual dietary diversity score (IDDS) can also be calculated by summing the number of food groups consumed by the respondent over the recall period using the nine food groups recommended by FAO (36, 37) and with scores ranging from zero to nine. IDDS represents an indicator of the nutrition quality of an individual diet. Extracted from the list of 20 possible food items in the questionnaire, the nine food groups included: (1) cereals/grains and root tubers; (2) vitamin A rich fruit and vegetables; (3) fruit other than vitamin A rich fruit; (4) vegetables other than vitamin A rich vegetables; (5) legumes and nuts; (6) meat, poultry, and fish; (7) oils and fats; (8) diary; and (9) eggs. Other items such as tea, sugars, and beverages were not considered when calculating the IDDS for this study. Independent variables including age, education, employment, household food consumption, region, and number of household members were also examined.

### Statistical analysis

Data were digitalised using CsPro during data collection and analyzed using SPSS version 26.0 (IBM Corp, 2019). Descriptive statistics and contingency tables were used to summarize household and participant characteristics and vitamin A rich food consumption. These were reported as proportions, percentages, means with corresponding standard deviations, and median values. The means and standard deviations for vitamin A consumption data for the women were computed from the weighted score, as well as separately for plant and animal sources of vitamin A. Exploratory factor analysis was conducted on knowledge items related to OFSP and summarized into two scales. For comparative statistics, bivariate correlations were conducted to examine associations between continuous independent variables and vitamin A rich food consumption. Multivariate linear regression tested the association between the outcome, vitamin A rich food consumption, and independent variables. Analysis of covariance was used to test associations between categorical variables. We considered a 95% confidence interval and statistical significance was set at *p* < 0.05.

## Results

Mean age was 28.3 ±6.9 and 73.2% of all respondents were in the 20–34 age category. Most of the respondents were married (88.1%) and 78.2% had seven or less years of formal schooling. A majority of the women either pregnant (16.8%) or lactating (79.6%) at the time of data collection. Most households (66.5%) had between 5 and 9 members and were engaged in farming. Seventy-seven percent of the women consumed four or fewer food groups in the week preceding the survey (Table 1).

**Table 1:** Socio-demographic characteristics of households and women.

**Variable**	**Categories**	***N* (%)**	**Mean (SD)**
**Study sub-region**	Eastern	216 (35)	
	Karamoja	137 (22.2)	
	Northern	19 (31.3)	
	West Nile	71 (11.5)	
**Household size**	1–4	127 (20.6)	6.6 (2.5)
	5–9	411 (66.5)	
	>9	80 (12.9)	
**Sex of household head**	Male	571 (94.4)	
	Female	34 (5.6)	
**Relationship to household head**	Household head	61 (10.5)	
	Spouse	494 (84.9)	
	Other	27 (4.7)	
**Mother characteristics**			
**Age group (yrs)**	15–19	44 (7.7)	28.3 (6.9)
	20–34	421 (73.2)	
	>35	110 (19.1)	
**Marital status**	Married with spouse	510 (88.1)	
	Married, spouse away	35 (6.0)	
	Divorced/separated	16 (2.8)	
	Widow	7 (1.2)	
	Never married	10 (1.7)	
**Education**	No formal education	138 (23.9)	4.99 (3.64)
(**yrs of schooling)**	1–7 years of schooling	321 (54.3)	
	>7 years of schooling	120 (20.8)	
**Main Occupation**	Farming (crops and livestock)	464 (80.3)	
	Household chores	62 (10.7)	
	Other	44 (8.3)	
**Physiological state**	Pregnant	97 (16.8)	
	Lactating	461 (79.6)	
	Non-pregnant, non-lactating	20 (3.5)	
**Disability**	Yes	22 (3.8)	
	No	557 (96.2)	
**IDDS**	≤ 4 food groups	443 (76.6)	3.4 (1.6)
	>4 food groups	135 (23.4)	

### Knowledge of vitamin A and orange-fleshed sweet potatoes

The majority of participants (82%) had heard about vitamin A and were able to identify the health benefits of the vitamin (Table 2). Almost a quarter of the women (22.7%) could identify the role of vitamin A in the prevention of infection and diseases. One fourth (24.6%) of the participants identified dark leafy green vegetables as a source of vitamin A, while only 3.1% identified OFSP as a source of vitamin A (Table 2). The mean score of vitamin A knowledge among participants was 2.1 ± 1.8 (Table 3). Based on a three-item scale, close to one third of the participants (30.7%) had no information about vitamin A and only 12% identified three sources and benefits of the vitamin (Table 3).

**Table 2:** Women's knowledge about vitamin A.

**Questions about knowledge on vitamin A**	**Yes (*n*)**	**%**
Heard about vitamin A	507	82
**Health benefits for vitamin A**		
Good for eye sight	69	11.2
Prevents infections/diseases	140	22.7
Important in blood production	10	1.6
Other benefits (i.e., healthy skin, appetite)	145	23.4
**Source of information about vitamin A**		
Health clinic	16	2.6
Media	27	4.4
Village health teams	26	4.2
School	17	2.8
**Identified sources of Vitamin A**		
Leafy green vegetables	152	24.6
Pumpkin/ripe mango/papaya	25	4.4
Orange-fleshed sweet potatoes	19	3.1
Eggs/Fish	132	21.4

**Table 3:** Vitamin A knowledge scores.

**Score**	**Frequency (%)**	**Mean score**
Score 0 (no information about vitamin A)	190 (30.7)	2.1 ± 1.8
Scored 1	69 (11.2)	
Scored 2	96 (15.5)	
Scored 3	91 (14.7)	
Scored 4	98 (15.9)	
Scored 5 (identified 3 sources and health benefits of vitamin A)	74 (12.0)	

Two factors, which informed the development of two scales presented in this analysis, were derived from factor analysis of the knowledge items on OFSP included in the questionnaire. The first factor was awareness of the general health benefits of eating OFSP and the second factor was misconceptions about OFSP (Table 4). Based on the identified OFSP knowledge scales, 45% of the participants scored above the mean of 2.46 ± 1.07 (median 2.5) in the knowledge scale for the general health benefits of OFSP and 61% scored below the mean score of 3.91 ± 0.82 (median 4) for misconceptions of OFSP (Table 5).

**Table 4:** Items and scale information from the exploratory analysis of knowledge about orange-fleshed sweetpotatoes included in the questionnaire.

**Item**	**Factor loading**				
	**1**	**2**	**1**	**2**	**3**
**General health benefits**					
Sweet potato leaves are good for human beings to consume	0.014	**0.726**	−0.066	**0.681**	0.365
Sweet potatoes that are orange inside are healthier than ones that are white inside	−0.027	**0.767**	−0.031	**0.8**	−0.266
**Misconceptions**					
Sweet potatoes are not good for the child	**0.723**	−0.23	**0.744**	−0.251	0.043
Sweet potatoes are not good for pregnant women	**0.816**	0.1	**0.837**	0.091	−0.063
Sweet potatoes are not good for lactating women	**0.771**	0.021	**0.749**	−0.032	0.302
Too much sweet potato can cause stomach problems	0.331	−0.229	0.309	−0.275	0.318
Vitamin A is found in all types of sweet potatoes	0.178	0.098	0.071	−0.009	0.848
**Eigenvalue**	1.94	1.23	1.94	1.23	1.07
**Percentage of variance**	27.6	17.5	27.6	17.5	15.3

The table indicates the factor loadings of the different items, when 2 and 3 factors were selected respectively. The bolded data indicates the correct items that loaded on a particular factor.

**Table 5:** Participant scores on the scale dimensions derived from factor analysis of knowledge of orange-fleshed sweet potatoes (OFSP).

**Scale component**	**Above mean *n* (%)**	**Below mean *n* (%)**	**Mean**	**Median**
General health benefits of OFSP	242 (45)	296 (55)	2.46 (1.07)	2.50
Misconceptions about OFSP	217 (37.9)	355 (61.3)	3.91 (0.82)	4.0

### Vitamin A rich food consumption for women

Based on the Hellen Keller International (HKI) guide (32), plant sources had the highest mean consumption in days per week compared to animal sources of vitamin A (Table 6). Foods presented in Table 6 only reflect vitamin A rich foods that were consumed at least once in the past seven days (frequency). Other foods listed in the HKI guide were consumed less than once in the past 7 days in this sample. Dark green leafy vegetables were the most common source of vitamin A for the study population, with a mean consumption of 2.84 days per week. OFSP were consumed at least once in the previous week by approximately 38% of the study population. Carrots, ripe mango, passion fruit or other vitamin A rich fruits, butter, and vitamin A fortified margarine were not commonly consumed by this sample. When consumed, animal sourced foods had the lowest frequency of consumption (Table 6), including small dried fish which was captured under the “any fish” category. There was a statistically significant difference in the mean number of days per week of consumption of plant (4.5) vs. animal (1.5) sources of vitamin A (*t, df* = −20.3, 616 *p* < 0.01). Nearly all women (>95%) had ≤6 days per week for the mean frequency of total consumption of animal and plant sources of vitamin A (weighted score). Based on the HKI guide (32), a community is considered to be at high risk of VAD when the mean weighted score is <6 days per week or <4 days of consumption of animal food sources of vitamin A.

**Table 6:** Vitamin A rich food group groups consumed at least once during the last week and meal frequency for women.

**Food groups**	***N* (%)**	**Mean frequency of consumption (days/wk)**
Any dark green leafy vegetables	450 (78.3)	2.84
Carrots	6 (1)	2.83
Ripe mango	46 (8)	2.32
Pumpkin or orange squash	153 (26.6)	1.9
Ripe pawpaw, fresh or juice	118 (20.5)	2.27
Passion fruit (or other fruit rich in vitamin A)	29 (5)	3.13
Orange-fleshed sweet potato	218 (37.9)	2.25
Eggs with yolk	110 (19.1)	1.9
Any fish, fresh	210 (36.5)	2.42
Liver from any animal	47 (8.2)	1.27
Butter	17 (3)	2.1
Vitamin A fortified margarine	8 (1.4)	2.25

### Predictors of vitamin A rich food consumption among women in Uganda

In the unadjusted regression model, knowledge of vitamin A (correlation coefficient −0.15, *p* < 0.01) and knowledge of the benefits of OFSP (correlation coefficient 0.10, *p* = 0.02) were significantly associated with women's vitamin A rich food consumption while misconceptions about OFSP was not (Table 7). Given the inverse correlation between vitamin A knowledge and vitamin A rich food consumption observed in the unadjusted model, we examined the data separately for correlations between vitamin A knowledge and plant vs. animal sources of vitamin A. Vitamin A knowledge had a statistically significant inverse coefficient (correlation coefficient −0.16, *p* < 0.01) with vitamin A rich animal sources and a non-significant correlation with vitamin A rich plant sources (correlation coefficient 0.03, *p* = 0.47).

**Table 7:** Estimated correlation coefficient and unstandardized (b) coefficient for association between independent variables and vitamin A rich food consumption for women.

	**Unadjusted model**	**Full Model** **Model 1**
**Variable**	***r*** **(coefficient)**	**B (SE)**
Household size	0.52	−0.04 (0.04)
Education	0.05	0.05 (0.03)
Age	0.06	−0.008(0.01)
Knowledge of vitamin A	−0.15^**^	−0.18 (0.50)^§^
General Knowledge of benefits of orange-fleshed sweetpotatoes	0.10^*^	0.18 (0.09)
Misconception about orange-fleshed sweetpotatoes	0.65	0.20 (0.11)
Individual dietary diversity score	0.07	0.78(0.11)

* Significant at *p* < 0.05,

** significant at *p* < 0.01,

§ significant at *p* < 0.001.

Multivariate linear regression analyses found that knowledge of vitamin A was significantly associated with women's vitamin A rich food consumption, after controlling for household size, education, age, and individual dietary diversity score, knowledge of OFSP, and misconceptions about OFSP [b (SE) = −0.18 (0.50)*, p* < 0.001] (Table 7). Neither knowledge of OFSP nor misconceptions about OFSP were independent determinants of vitamin A food consumption (Table 7).

## Discussion

Our findings contribute to the literature on predictors of vitamin A rich food consumption. Based on the Hellen Keller assessment guide (32), the study population was found to be at a high risk for vitamin A deficiency given that the consumption patterns of vitamin A rich foods for women were relatively low with a mean weighted score below the threshold of adequate consumption. This is compounded by findings that suggest generally low knowledge about vitamin A was observed in this sample where a significant proportion of women were either pregnant or lactating at the time of data collection. These stages of the life course have higher vitamin A requirements and low intakes during these stages are associated with adverse health consequences (38). Recommendations are based on the expected secretion of retinol into human breast milk which is dependent on the mothers' vitamin A status, with the expectation that infants would also benefit (39). Breast milk is a fundamental source of vitamin A, especially in the first 6 months of life (40). Given the critical role of vitamin A in infant health and development, it is essential that women of reproductive age get adequate vitamin A and other micronutrients (41).

The targeted regions are post conflict areas of Uganda that experience greater food and nutrition insecurity due to food shortages and disrupted economic activities. In Uganda, like in many other developing countries, large scale programs are needed to make a significant impact on high rates of malnutrition, including VAD. However, there has been limited experience and success in scaling up programs (42). Based on the weighted consumption score, which found that nearly all women consumed low amounts of both animal and plant sources of vitamin A, the surveyed communities are considered to be at a high risk for VAD. Plant foods were the most frequent sources of vitamin A compared to animal sources of vitamin A which influences vitamin A status due to bioavailability of carotenoids (43).

The vitamin A rich food consumption patterns of the participating communities were not uniquely different and accentuated the reliance on plant source diets in limited resource settings in Sub Saharan Africa. Animal source foods are associated with higher serum retinol levels (44), however, are less affordable for low income populations (45). Previous studies corroborate our findings, suggesting that individuals from resource-constrained settings have limited access to foods containing preformed vitamin A from animal-based food sources and they do not commonly consume available foods containing beta-carotene due to poverty or lack of information on the importance of the food sources (46).

Nutrition education has the potential to increase knowledge of nutrition benefits among at-risk communities to improve consumption of carotenoids, including of OFSP, mangoes, papaya and dark leafy green vegetables and diversification to include animal sources of vitamin A (47). However, we observed a significant and inverse association between knowledge of vitamin A and vitamin A rich food consumption, after adjusting for covariates. This suggests that despite gains in nutrition knowledge, other barriers may play a role in the levels of consumption of vitamin A rich foods in these regions of Uganda. Some consumption barriers could include lack of access of vitamin A rich foods due to seasonal variation, especially among small holder subsistence farmers who are commonly stricken by poverty (48).

Seasonal variation in food supply can be addressed by growth of drought-resistant and dry season crops, such as tubers, which can alleviate food insecurity, although they may not necessarily be the best sources of vitamin A (49). Leafy green vegetables and OFSP were the commonly consumed sources of vitamin A in this population. Being seasonal crops, this translates into reduced consumption during out of season periods. Seasonality needs to be considered for consistent vitamin A intake across the year. Having the potential to purchase or grow other sources in the market during off seasons is a strategy to be considered in program interventions. Education and peer mentoring on home-based gardens for alternative vitamin A sources during periods of food insecurity, especially in the dry season, could prove to be instrumental in mitigating malnutrition (50, 51).

Vitamin A rich plant foods could also be readily available in the community but likely underutilized. In these situations, existing cultural practices that prohibit pregnant and lactating women from consuming potentially good sources of vitamin A may pose another barrier to consumption that may need to be abated (52, 53). Targeted nutrition education can be incorporated in food-based programs to increase community knowledge on ways to improve health outcomes, including bioavailability and absorption of vitamin A from plant sources (42, 54). Education can include preparation methods that allow the addition of fat to increase bioavailability of vitamin A which is fat soluble and can be stored in the body (55). An intervention study, with nutrition education, conducted among adolescent girls in Sri Lanka resulted in a highly significant (*P* < 0.001) increase in knowledge and consumption of local vitamin A rich foods (56). The percentage of adolescent girls with low serum retinol concentrations (<20 microg/dL) decreased from 17 to 4.8% as a result of the intervention (56).

Similar to this study, low dietary diversity was highly prevalent among pregnant women (*N* = 104) in the Damot Sore district of Southern Ethiopia (57). Another studies in Ethiopia reported that one quarter of households had low dietary diversity (58) or low to medium dietary diversity (20). In contrast, a study among 624 lactating mothers attending an immunization clinic in Gondar town, Ethiopia, determined that women had adequate vitamin A consumption with a greater proportion of women consuming more than five food groups (20). Nutrition knowledge was also found to be higher among women attending an immunization clinic in Gondar town, Ethiopia (20).The difference in vitamin A rich food intake in these Ethiopian communities might be explained by the women in Gondar having access to health care, experiencing greater food security, and residing in a semi-urban area of the country.

We found that knowledge of vitamin A was a statistically significant predictor of vitamin A rich consumption after adjusting for covariates. Similarly, knowledge was a predictor of vitamin A rich food consumption in a Nepali study (59). However, no statistically significant association was found between knowledge of vitamin A and vitamin A rich food consumption among lactating women in two regions of Tanzania (60). While age, education, household size, and individual dietary diversity were not predictors of vitamin A rich food consumption in this study, other studies suggest otherwise (21, 60, 61). Having a college degree, family size, and being from a higher economic class were identified as factors associated with vitamin A consumption in an Ethiopian study (20). Given that the study was conducted in an urban setting, it is possible women who attended the clinic in this town setting were more educated, knowledgeable, and economically well off (20) compared to women in our study who lived in post-conflict areas and relied on farming income. A study conducted in northern Benin where 34% of women were identified as being at a greater risk of VAD, found that maternal education, maternal farming activity, maternal health status, low food diversity, lack of fruit and vegetable consumption, low protein food consumption, and high infection rates were associated with the vitamin A status (61). In a study of 569 lactating mothers in Tanzania, the prevalence of VAD was 88.5% and was associated with place of residence (60). Sixty-eight percent of the lactating mothers in this Tanzanian study lacked knowledge about vitamin A and fortified oil, however they had a positive attitude toward the consumption of vitamin A rich foods (60). These findings suggest benefits of nutrition education, specifically on vitamin A and its health benefits, in low-resourced communities (60).

Our study contributes to scientific knowledge on predictors of vitamin A rich food consumption in low resource rural settings with subsistence farming activity. The results of this study will inform the International Potato Center's (CIP) implementation and scaling up of initiatives related to OFSP in selected post conflict regions in Uganda; adjustments to the initiative that improve nutrition education and remove consumption barriers can promote program effectiveness related to improved vitamin A rich food consumption within these communities.

By the time this paper was written, scaling up the program for the delivery of biofortified OFSP had begun to improve accessibility which may have translated into higher consumption among participating communities.

CIP is partnering with humanitarian agencies such as the World Food Programme (WFP) to make OFSP available for improving nutrition and livelihoods of vulnerable populations. This project is built on the premise that the proven nutrition and livelihood benefits of OFSP can be delivered efficiently through existing large-scale humanitarian programs, especially because these transition into market-based approaches that engage local and national agri-food systems to respond to and reduce the need for humanitarian food aid. Sweet potatoes and specifically OFSP, is new in the farming and food systems of the targeted regions. The intervention with the introduction of OFSP in these drought-prone post conflict regions has shown that the crop is an important part of the local diet and food system, where roots and leaves are both consumed as part of a healthy diet. The results of this study provide the CIP with important lessons on barriers to consumption, opportunities to improve uptake, and ultimately on strategies for scaling up of OFSP to improve the nutritional status, livelihoods, and food security status of these vulnerable populations.

## Limitations

Limitations include the lack of serum level data for assessment of vitamin A status of our participants. Although vitamin A consumption was computed from the Hellen Keller International guide which has been validated against acceptable standards, it is still possible underestimation of vitamin A consumption may have occurred based on the vulnerable population of women and the local environment. In addition, responses to the questionnaire could have been prone to both recall and social desirability bias. Finally, food practices/patterns could be unique to the post conflict situation of the rural areas of Uganda. Results may not be generalizable to populations in other regions or urban areas of Uganda.

## Conclusion

We found that the study population was considered to be at high risk for VAD. Knowledge of vitamin A rich foods was an inverse and significant predictor of vitamin A rich food consumption among women, suggesting other barriers to uptake of these foods. Components of food insecurity such as availability, affordability, utilization, and changing food preferences may contribute to the unexpected inverse relationship between knowledge and consumption of Vitamin A rich foods. Scaling up biofortified food initiatives, including OFSP, can improve consumption of vitamin A rich foods with effective strategies to comprehensively address consumption barriers such as lack of nutrition education, cooking skills, and storage facilities, as well as low production levels and perceived contamination of biofortified foods. The results of the study suggest that food-based interventions addressing vitamin A rich food consumption through scaling up of biofortified foods, such as orange-fleshed sweet potatoes, consider opportunities to address the broad range of consumption barriers that persist despite gains in nutrition knowledge.

## Data availability statement

The raw data supporting the conclusions of this article will be made available by the authors, without undue reservation.

## Ethics statement

The study involving human participants was reviewed and approved by the Makerere University School of Health Sciences Institutional Review Board and received a waiver from the University of Massachusetts Amherst subject protection committee. Written informed consent from the participants' legal guardian/next of kin was not required to participate in this study in accordance with the national legislation and the institutional requirements.

## Author contributions

JN and LC drafted the manuscript. JN, LC, LS, FG, NK, and SH contributed to the conception of the study. FG, NK, and SH collected the data and were responsible for data preparation. LC, LS, EM, and FG reviewed the manuscript and critically revised it for important content. SH assisted with additional research resources. All authors read and approved the final manuscript.
