# Supplementary material for: Predictors of vitamin A rich food consumption among women living in households growing orange-fleshed sweetpotatoes in selected regions in Uganda
Source: Front Public Health. 2023 Jan 9;10:880166. doi: 10.3389/fpubh.2022.880166 (PMC9868160; doi:10.3389/fpubh.2022.880166)
Supplement: Supplementary file 1 [file Data_Sheet_1.PDF]

SUPPLEMENTARY MATERIAL: KNOWLEDGE QUESTIONS ON THE SURVEY

PART J. KNOWLEDGE, ATTITUDE AND PERCEPTIONS OF VITAMIN A (RESPONDENT IS REFERENCE MOTHER AND OR CHILD CAREGIVER ONLY)

**j.1**	Have you ever heard of Vitamin A? **1**=Yes **0**=No >>>*go to* ***6***	
**j.2**	If Yes, how long ago did you first hear of Vitamin A?<br>**1**=Recently; **2**=1-3 months; **3**=4- 6 months; **4**= 6-12 months; **5**=1-2 years; **6**=More than 2 years; 77= don't know/remember; 99=NA	
**j.3**	If Yes, tell me 2 main reasons why Vitamin A is important<br>**1**=Prevents disease; **2**=Good for eye sight; **3**=Keeps skin healthy; **4**=Produces red blood cells/keeps blood healthy; **77**=Don't know; 99=NA	
**j.4**	Where did you learn about Vitamin A? (code any three mentioned in the list)<br>**1**=Vernacular radio; **2**=English radio; **3**=School child **4**=VHT **5**=Friend/relative; **6**= When I was still in school ; **7**=Health clinic; **8**= print media 9=Other; 99=NA	
**j.5**	Please name any t**hree** examples of foods that are rich in Vitamin A<br>**1**=Ripe mango; **2**=Ripe papaya; **3**=Carrot; **4**=Eggs; **5**=Pumpkin; **6**=Liver; **7**=Red pepper; 8=mention of dark green vegetable **9**=Other.............; 99=NA	
**j.6**	Do you ever purchase sweetpotato for home consumption? **1**=Yes; **0**=No>>> go to **13**	
**j.7**	If Yes, when was the last time you purchased sweetpotato for home consumption?<br>**1**=few days ago; **2**=few (2-4) weeks ago; **3**=few (2-5) months ago; **4**=more than 6 months ago;	
**j.8**	If you purchased sweetpotato for home consumption, what type was it?<br>**01**=White flesh; 0**2**=Yellow flesh; 0**3**=Orange flesh; **77**=don't know/can't remember	
**j.9**	What quantity did you purchase? [*Use unit code*].	
**j.10**	How much did you pay for this quantity?	(UGX)
**j.11**	Is the amount you paid OK? **1**=normal price; **2**=cheap; **3**= expensive	
**j.12**	If you had more money, how much OFSP would you eat compared to now?<br>**01**=Less; 0**2**=More; 0**3**=The same; **88**=Never;	
**j.13**	In your opinion, what is healthier to eat for breakfast? **1**=Sweetpotato; **0**=Bread	
**j.14**	Have you participated in vitamin A supplementation using a capsule **1**=Yes; **0**=No	
**j.15**	When was the last time you received a supplication dose of Vitamin A capsule?<br>**01**=less than 1 month ago; 0**2**=1-3 months ago; 0**3**=4-6 months ago; 0**4**=6-12 months ago ;<br>**05**=more than 1 year ago; **77=**don't know ; **88**=Never	
j.16. Have you participated in any of the following …(questions 16a-16c)		
**j.16a**	Nutritional cooking demonstrations using OFSP? **1**=Yes; **0**=No	
**j.16b**	Nutrition education about using OFSP for feeding infants and young children **1**=Yes; **0**=No	
**j.16c**	Individual nutrition counseling program talking about the use of OFSP? **1**=Yes; **0**=No	
[*Enumerator please read*]: Now I am going to read you several statements related to sweetpotato and I would like you to rate each statement based on the scale below…		
**Scale: 01=strongly agree 02=Agree 03=I do not have an opinion 04=Disagree 05=strongly disagree 77=don't know/not sure**		

		Scale			Scale

**j.17a**	Sweetpotato leaves are good for human beings to consume		**j.17h**	I am proud to serve sweetpotatoes to my family	
**j.17b**	Sweetpotato that are orange inside are healthier than ones that are white inside		**j.17i**	You can't eat too much sweet- potato because you will get stomach problems	
**j.17c**	Sweetpotato is the most reliable food crop for our family during times of food shortage		**j.17j**	It does not pay to increase the area growing sweetpotato, because there is no market to sell it	
**j.17d**	Even when we have lots of maize, cassava, or potato to eat, we still like to have sweetpotato in our diet		**j.17k**	Sweetpotato should be promoted as an important crop by the relevant government of my county/country	
**j.17e**	You can't grow sweetpotato and be considered a man		**j.17l**	Vitamin A is found in all types of sweetpotato	
**j.17f**	Sweetpotato is not good for children less than 2 years old		**j.17m**	Sweetpotato is not good for lactating women	
**j.17g**	Sweetpotato is not good for pregnant women		**j.17n**	Sweetpotato is a woman’s crop	
